# Genome Analysis of *Lactobacillus plantarum* Isolated From Some Indian Fermented Foods for Bacteriocin Production and Probiotic Marker Genes

**DOI:** 10.3389/fmicb.2020.00040

**Published:** 2020-01-29

**Authors:** Aditi Goel, Prakash M. Halami, Jyoti Prakash Tamang

**Affiliations:** ^1^Department of Microbiology and Fermentation Technology, CSIR-Central Food Technological Research Institute, Mysuru, India; ^2^DBT-AIST International Centre for Translational and Environmental Research and Bioinformatics Centre, Department of Microbiology, School of Life Sciences, Sikkim University, Gangtok, India

**Keywords:** antimicrobial activity, reporter bacteria, plantaricin, bacteriocin, probiotics

## Abstract

In this study, *Lactobacillus plantarum* strain DHCU70 isolated from *dahi*, a fermented milk product and *L. plantarum* strain DKP1 isolated from *kinema*, a fermented soybean food of India, respectively were evaluated for their bacteriocin production and probiotic properties. Both strains of *L. plantarum* (DHCU70 and DKP1) were found to have potent antimicrobial activity against *Kocuria rhizophila* ATCC 9341. Bacteriocin produced by *L. plantarum* strains DHCU70 and DKP1 did not exhibit inhibition of cell wall, DNA and fatty acids biosynthesis mechanisms as evaluated by whole cell reporter assays. We characterized the bacteriocin encoding genes in *L. plantarum* strains DHCU70 and DKP1 by whole genome sequence which consisted of a single and circular chromosome with genome size of 3.38 Mb (GC content of 44.3%) and 3.39 Mb, respectively and a GC content of 44.3%. *L. plantarum* DHCU70 has 3252 number of protein encoding genes comprising 89 number of RNA genes (69tRNA, 16rRNA, 4nc RNA) whereas *L. plantarum* DKP1 has total of 3277 number of protein encoding genes with 89 number. of RNA genes (69tRNA, 16S rRNA, 4nc RNA). Analysis revealed the presence of 20.5 kb long and 23 numbers of plantaricin encoding locus (*pln* locus) for production of antimicrobial compound. BAGEL analysis has shown that the *pln* locus of both the strains of *L. plantarum* showed maximum sequence similarity with plantaricin NC8 of *L. plantarum* NC8, originally isolated from grass silage. Annotated whole genome sequence of both strains DHCU70 and DKP1 was analyzed for the presence of probiotic marker genes. The probiotic properties of these strains of were also evaluated *in vitro*. Due to the presence of genes responsible for antimicrobial activity and probiotic properties, both strains of *L. plantarum* may be considered as a suitable probiotic candidate in food industry.

## Introduction

Fermented foods and beverages have several functional microorganisms including many species of lactic acid bacteria (LAB) which are well known for antimicrobial and probiotic properties (Tamang et al., 2016b; Rezac et al., 2018). LAB are certified as generally regarded as safe (GRAS) bacteria granted by the American Food and Drug Agency (FDA)^1^. Most of the genera belong to LAB such as *Lactococcus, Lactobacillus, Leuconostoc, Pediococcus*, and few *Streptococcus* have also received the Qualified Presumption of Safety (QPS) status by European Food Safety Authority (EFSA)^2^. LAB mostly act as protective cultures due to the production of antimicrobial compounds bacteriocin, nisin, enterocin, etc. (Gaggia et al., 2011; Tamang et al., 2016a) which are present in many fermented dairy and vegetable (Tamang et al., 2009; Jiang et al., 2012; Grosu-Tudor and Zamfir, 2013; Mokoena, 2017; Silva et al., 2018). Among the various species of *Lactobacillus*, *L. plantarum* is well known for its probiotic application due to its natural habitation in human gastro intestinal tract (Zhang et al., 2012) and has several functional properties such as antioxidant activity, anti-cholesterol effect, bio-protective and immune-modulation (Ren et al., 2014; Devi et al., 2015, 2016; Tamang et al., 2016a). *L. plantarum* has both narrow and broad spectra of antibacterial activity against Gram-positive pathogenic bacteria viz *Listeria monocytogenes*, *Staphylococcus aureus* and also against Gram-negative pathogenic bacteria such as *Salmonella Typhimurium*, *Klebsiella, Rhizophila, E. coli, Aeromonas hydrophila*, and *Yersinia* (Behera et al., 2018; Silva et al., 2018; Spangler et al., 2019).

*Lactobacillus plantarum* produces antimicrobial compound known as plantaricin (Song et al., 2014), which has a bactericidal mode of action against other closely related microorganisms by dissipating the proton motive force or by creating the pores in the cell membrane that permits the efflux of relatively large molecules (Todorov, 2009). Most of the experiments on the mode of action is being done by conventional microbiological methods involving screening of antimicrobial compound by agar well diffusion assay, which are tedious and unwieldy (Balouiri et al., 2016). Nowadays, the application of high-throughput screening (HTS)with targeted cell-based assays, that carry reporters such as β-galactosidase or luciferase genes (Shobharani and Halami, 2016) for microbial compounds, is getting more popular and reliable (Baker et al., 2016).

Different strains of *L. plantarum* are genetically diverse with respect to genome size, number of proteins and diversity in plantaricin encoding locus (Devi and Halami, 2019). The genome size of *L. plantarum* varies from 3.0 to 3.3 Mb (Li et al., 2015). *L. plantarum* P8 (Accession no. NC_021224) has genome size of 3 Mb with total no of 2892 protein encoding genes whereas *L. plantarum* WCFS1 has genome size of 3.3 Mb with 3057 number of total protein encoding genes (Accession no. NC 004567). *L. plantarum* ZJ316 (Accession no. NC_020229) found to have 3159 number of protein encoding genes. Devi and Halami (2019) characterized the plantaricin produced by *L. plantarum* in different plantari-types based on presence or absence of various *pln* genes. *Pln* locus of plantaricin encoding genes is genetically organized in either simple or complex operon (Diep et al., 2009). Plantaricin 423, S and W are found on one simple operon (Nissen-Meyer et al., 2009) whereas, *L. plantarum* C11, WCFS1, JDM1, J23, J51 and NC8 strains are organized in complex *pln* locus consisting of 25–28 genes in the mosaic-like structure of 5–6 operons encoding Class IIb bacteriocin (Diep et al., 2009; Tsapieva et al., 2011; Tai et al., 2015). All bacteriocin related information collected from bacteriocin databases, that have been created to compile the increasing number of bacteriocins characterized from both Gram-positive and Gram-negative bacteria, may be used for the automated screening of bacteriocin gene clusters (Blin et al., 2013; Van Heel et al., 2013).

Some species of *Lactobacillus* isolated from ethnic fermented foods of Sikkim in India were reported to have antimicrobial activities (Tamang et al., 2005, 2008, 2009; Dewan and Tamang, 2007; Tamang and Tamang, 2009). However, there has been no report of whole genome sequencing of *Lactobacillus* species, isolated from ethnic fermented foods of Sikkim in India, targeting the bacteriocin producing gene for characterization of bacteriocin and identification of probiotic genes. The present work aims to study the whole genome sequence of *Lactobacillus* strains isolated from some ethnic fermented foods of Sikkim, India to characterize the bacteriocin producing gene and evaluation of its probiotic properties.

## Materials and Methods

### Bacterial Strains and Culture Condition

Different strains of LAB were isolated from two common ethnic fermented foods of Sikkim in India viz. *kinema*, fermented soybean food (Tamang, 2015) and *dahi*, fermented milk product (Rai et al., 2016), respectively. Isolates were sub-cultured in de Man, Rogosa and Sharpe (MRS) media (HiMedia, Mumbai, India) and incubated at 37°C for 18 h. *Kocuria rhizophila* ATCC 9341, used as an indicator strain to check the antimicrobial activity of the isolates, was cultured in BHI broth (HiMedia, India) and incubated at 37°C for 24 h under aerobic conditions. Study on mode of action was carried out by using reporter strains of *Bacillus subtilis* BSF2470 (cell wall inhibition), AUT1 (Nisin specific reporter), *yor*B promoter (DNA inhibition) and *fab*HB (fatty acid inhibition) (Shobharani et al., 2015), which were stored at −20°C in Luria Bertani (LB) broth containing 16% (v/v) glycerol following the method of Nithya and Halami (2012). Reporter cultures were sub-cultured to antibiotics (erythromycin at a concentration of 5 mg/ml for BSF and chloramphenicol at a concentration of 5 mg/ml for all other reporters) containing fresh LB broth and incubated aerobically at 37°C, 150 rpm for 24 h (Shobharani et al., 2015).

### Evaluation of the Antimicrobial Activity of *L. plantarum*

Previously isolated bacterial isolates from various fermented foods of North East India was evaluated for bacteriocin production by agar well diffusion method (Li et al., 2015). Cells were grown in MRS for 16–18 h and then centrifuged at 8000 rpm, 4°C for 15 min to remove the cells. The supernatant was neutralized to the pH of 7.0 with 0.1 N NaOH followed by filter-sterilization through 0.2 μm membrane. Consequently agar well diffusion assay was performed against indicator strain *K. rhizophila*. Briefly, 50 μL of cell free supernatants were placed into 6 mm wells on BHI agar plates seeded with the above indicator strains. After incubation at 37°C for 12 h, the diameters of inhibition zones were measured. Proteinaceous nature of antibacterial substance was checked by incubating the cell-free supernatant (CFS) with 1 mg/ml of proteinase K (Hi Media) at 37°C for 2 h (Devi and Halami, 2011). Both protease-treated CFS were assayed for activity as indicated above (Devi and Halami, 2011). CFS was also tested for heat resistance at boiling temperature for 15 min (Sure et al., 2016).

### Characterization of Isolates

Isolates of LAB (DHCU70 strain from *dahi* and DKP1 strain from *kinema*) showing prominent anti bacterial activity against *Kocuria rhizophila* were characterized by using conventional methods involving Gram-staining, SEM analysis followed by various Biochemical tests – Catalase Test, Growth at various temperature, pH and salt concentrations(Li et al., 2015). Sugar fermentation profile was checked by incubating the cultures at 37°C for 48 h in Bromo Cresol purple broth with different sugars as described by Schillinger and Lücke (1987). Identity of cultures was confirmed by 16S rRNA gene sequencing using one set of bacterial universal primers 27F (5′-AGAGTTTGATCCTGGCTCAG-3′) and 1492R (5′-GGTTACCTTGTT ACGACTT-3′) (Lane, 1991). Subsequently, taxonomy of the cultures was confirmed by whole genome sequencing analysis.

### Chromogenic Plate Assay for the Mode of Action

Study on mode of action was carried out by using whole cell reporter assay (Nithya and Halami, 2012). Briefly, overnight grown reporter bacteria in antibiotic containing LB Broth was sub-cultured again in LB Broth without antibiotic and incubated at 37°C aerobically till the absorbance reached to 0.7 OD_600_. Subsequently, LB agar plates containing reporter bacterial strain supplemented with 50 μg/ml of X-Gal were prepared. Then isolates were spotted on the LB agar plate and incubated for 24–36 h at room temperature until a blue coloration was observed due to the induction of *lac*Z resulting in the production of β-galactosidase.

### DNA Extraction

Extraction and purification of genomic DNA of isolates was carried out using Qiagen DNeasy blood and tissue kit (Qiagen, Hilden, Germany). The concentration and purity of genomic DNA was quantified by previously calibrated Nano-drop spectrophotometer (Thermo Fisher Scientific, Waltham, MA, United States) and Qubit fluorometer (Invitrogen, United States).

### Genome Sequencing

Whole genome sequencing of two tentatively identified *L. plantarum* cultures: DHCU70 and DKP1 was carried out following the method of Tanizawa et al. (2015) using Illumina Miseq 300 × 2 Platform (Illumina, San Diego, CA, United States) at the facility of Genotypic Technology Pvt. Ltd., Bangalore. A total number of 55143 and 567922 reads were obtained for *L. plantarum* DHCU70 and DKP1, respectively, with N50 value 135,312 and 135,926, respectively.

### Bioinformatics Analysis

High-quality reads were assembled in contigs using SPADES 3.9.1 assembler. Later the order of contigs was determined by aligning the contigs with the genome sequence of originally published strain *L. plantarum* Wcfs1 (GenBank Accession no. AL935263). Gene prediction for assembled genome was carried out using Genmark which were annotated by similarity searched against UniProt bacterial protein database using DIAMOND BLAST with an e-value of1e-5 for gene ontology and annotation (Buchfink et al., 2015). Prophage insert regions were detected with an on-line phage search tool, PHASTER (Arndt et al., 2016). The CRISPR regions were identified with a CRISPR on-line detection tool, CRISPR finder (Grissa et al., 2007). Information about plasmids was obtained by using online tool Plasmid Finder.

### Phylogenetic Analysis

The genetic presence and comparison of 16S rRNA and *rec*A genes were performed by analyzing the nucleotide sequence data available at National Center for Biotechnology Information (NCBI) database. The 16S rRNA and *rec*A gene sequences of related organisms were obtained from NCBI database and compared with the sequences of our *L. plantarum* strains to know the closest neighbor in the evolutionary tree. Neighbor-joining (NJ) phylogenetic tree with p-distance model was constructed by MEGA 6 software (Tamura et al., 2011).

### Identification of Bacteriocin Encoding Genes

Different *pln* genes were identified in WGS of *L. plantarum* strains DHCU70 and DKP1 by sequence similarity search using BLASTP and compared with already known plantaricin (Diep et al., 2009). Subsequently, organization of plantaricin encoding genes of individual strain of *L. plantarum* was analyzed using bacteriocin database BAGEL4 (Van Heel et al., 2013).

### Probiotic Functionalities

#### Identification of Probiotic Genes

The sequence information for different probiotic genes (Lebeer et al., 2008) of related strain was obtained from the NCBI database and used to find out the probiotic genes present in our *L. plantarum* strains by sequence similarity search using BLASTP.

#### Acid and Bile Salt Tolerance

Acid and bile tolerance of *L. plantarum* cultures was evaluated as described by Archer and Halami (2015). For acid tolerance MRS broth was adjusted to pH 3 with 1N HCl; and for bile salt tolerance test, 0.3% (w/v)bile salts (MP Biomedicals, India Pvt. Ltd.) was added to MRS broth. The broth with adjusted pH values and bile salt concentration were inoculated with 10^9^CFU/mL of O/N grown cultures of *L. plantarum* DHCU70 and DKP1 strains and incubated at 37°C. Each tube containing 1 ml of culture was taken at 0, 1, 2, 3, and 24 h interval and absorbance was measured at 600 nm. Subsequently cultures were serially diluted in 0.8% saline water and plated on MRS agar followed by incubation at 37°C for 48 h. The viable bacterial cell counts in terms of the colony forming units (cfu/ml) were recorded after 24 h. All the experiments were repeated twice. MRS broth with neutral pH 7.0 and without bile was served as a control, respectively.

#### DPPH Radical-Scavenging Assay

Antioxidant activity was measured by DPPH assay as described by Son and Lewis (2002) and Archer and Halami (2015). Briefly overnight grown cultures were centrifuged at 8000 rpm for 10 min at 4°C and the CFS was collected. 100 μl of CFS was mixed with 1.9 ml of methanol. Later, 2 ml of 2,2-diphenyl-1-picrylhydrazyl (DPPH) (6 mg/100 ml of methanol)was added to CFS. DPPH without addition of CFS was used as control while only methanol was used as blank. The tubes were mixed properly and incubated at room temperature for 30 min in dark. After incubation absorbance was measured at 517 nm and DPPH activity was measured by following formula:

DPPH⁢radical⁢scavenging⁢activity%=[(Acontrol-Atest)/Acontrol]×100

#### Cell Surface Hydrophobicity

Bacterial adhesion to hydrocarbons was determined according to the method described by Rosenberg et al. (2006). Bacterial cells were grown in MRS broth at 37°C for 18 h and centrifuged at 8000 rpm for 10 min. The cell pellets were washed twice with phosphate buffer, pH 7.0, resuspended in phosphate buffer and the initial absorbance was adjusted to 0.7 OD at 600 nm (Ab_i_). Cell suspension was mixed with n-hexadecane or xylene (3:1), vortexed and incubated at 37°C for 10 min. The mixture was again vortexed and kept at 37°C for 1 h for phase separations. The aqueous phase was removed gently and its absorbance (Ab_F_) was measured at 600 nm. The surface hydrophobicity (%) was calculated as per the following formula:

Surface⁢Hydrophobicity=100×(AbI-AbF)/AbsInitial

#### Cellular Auto-Aggregation

Auto-aggregation was performed as described by Archer and Halami (2015). The 5 ml of cultures were mixed properly and incubated at 15°C for 2–3 h. One ml of upper suspension was taken from undisturbed incubated tube; OD was measured at 600 nm and percentage auto aggregation was calculated as follow:

Percentage⁢aggregation=1-(OD⁢of⁢upper⁢suspension/OD⁢of⁢total⁢culture)×100

## Results and Discussion

### Evaluation of Antimicrobial Activity

**FIGURE 1:**
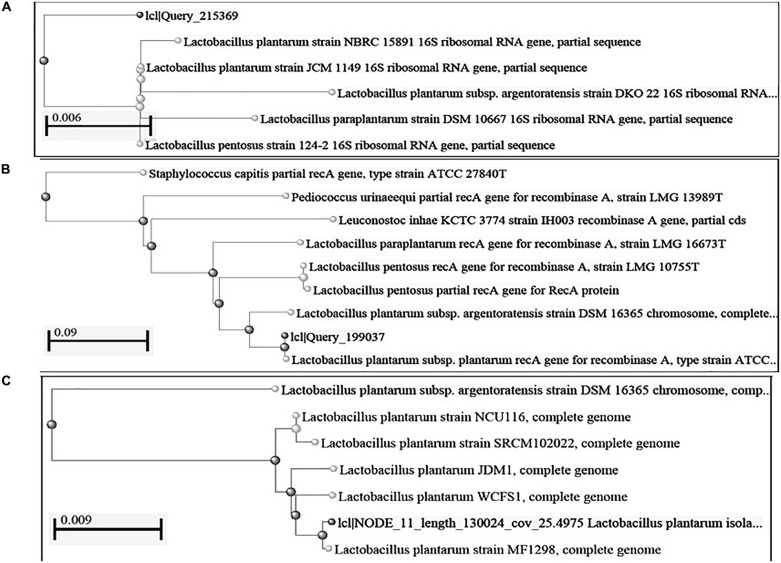
Construction of phylogenetic tree using NCBI Database by Neighbor-Joining method **(A)** 16S rRNA gene; **(B)**: *rec*A gene sequence; **(C)**: complete genome sequence.

We isolated 683 bacterial isolates from various fermented foods of North East India, out of which 129 lactic bacteria bacterial isolates were found to produce an antimicrobial compound (data not shown). All 129 isolates were phenotypically characterized on the basis of physiological and biochemical tests out of which, only seven isolates were tentatively identified as *Lactobacillus plantarum*. Out of seven strains of *L. plantarum*, only two strains- DHCU70 isolated from *dahi*, a fermented milk product of Sikkim and DKP1 isolated from *kinema*, a fermented soybean food of Sikkim, India, showed prominent inhibition zones against *Kocuria rhizophila* ATCC 9341for untreated CFS, CFS at acidic pH, CFS at basic pH and CFS treated at boiling temperature (Supplementary Table S1 and Supplementary Figure S1). Antimicrobial activity of both strains of *L. plantarum* was found to be stable at boiling temperature, acidic (pH 3) and basic (pH 9) (Supplementary Table S1 and Supplementary Figure S1). Identification of *Lactobacillus plantarum* strains DHCU70 and DKP1 was confirmed by 16S rRNA gene sequences (Figure 1A). Antimicrobial compound produced by *L. plantarum* strains DHCU70 and DKP1 was found to be proteinaceous in nature, as when CFS was treated with proteinase K it showed complete loss of antimicrobial activity (Yang et al., 2012).

Similar observations were earlier reported on broad spectrum of activity of plantaricin LR14 produced by *L. plantarum* against *Kocuria rhizophila, Listeria monocytogenes, Salmonella, Yersinia, enterocolitica, Bacillus lichniformis*, and *E. coli* (Tiwari and Srivastava, 2008). Plantaricin LPL-1 isolated from fermented fish was active against *S. aureus, Listeria. monocytogenes, B. pumilus, B. amyloliquefaciens, E. faecalis, L. plantarum, L. delbrueckii, L. bulgaricus, L. salivarius*, and *L. lactis* (Wang et al., 2018). *L. plantarum* strains isolated from fermented vegetables products of India showed antibacterial activity against *K. rhizophila* (Devi and Halami, 2019). Besides, antibacterial activity plantaricins also showed thermo-stability (Behera et al., 2018; Lim et al., 2018).

**FIGURE 2:**
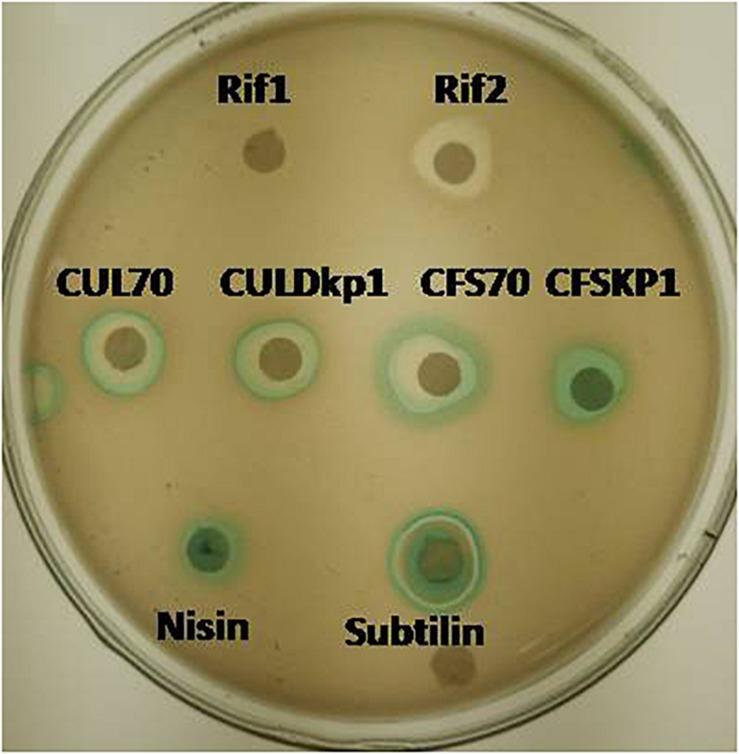
Mode of action studies using whole cell reporter assay for *Lactobacillus plantarum* strains DHCU70 and DKP1. Indicators: Rif1-Rifampicin (–ve control) 1 μg/ml, Rif2-Rifampicin (–ve control) 0.1 μg/ml, CUL70- culture of dhcu70, CULDKP1- culture of DKP1, CFS70 – Cell filtrate supernatant of DHCU70, CFSDKP1 – cell filtrate supernatant of DKP1; Nisin and Subtilin (+ve control).

The mode of actions was carried out by using whole cell reporter assay. *L. plantarum* strains DHCU70 and DKP1 induced the production of β-galactosidase in reporter strains of *Bacillus subtilis* BSF2470 specific for cell wall inhibition (Figure 2). Bacteriocin produced by DHCU70 and DKP1 strains of *L. plantarum* did not show any positive response toward DNA specific reporter (*yor*B) as well as fatty acid specific reporter (*fab*HB) indicating that bacteriocin produced by both the strains of *L. plantarum* did not act on DNA and fatty acids (data not shown). So it may be concluded that bacteriocin produced by DHCU70 and DKP1 acts through inhibition of cell wall. In general, class II bacteriocins show bactericidal mode of action by dissipating the proton motive force by disrupting the trans-membrane potential or pH gradient of indicator strains (Todorov, 2009). Specifically in class IIb bacteriocins, two peptide bacteriocin acts by creating pores which results in dissipating trans membrane potential (Zhang et al., 2015). As most of the *L. plantarum* known till date found to act on cell membrane (Arena et al., 2019). Interestingly, *L. plantarum* strains DHCU70 and DKP1 did not show positive result on *Bacillus subtilis* BSF2470 reporter. So we repeated the assay with concentrated bacteriocin preparation and observed that the AMC produced by *L. plantarum* strains are acting on BSF2470 reporter (Figure 2). These results found to be consistent in every repetition.

### Genome Analysis of *L. plantarum*

**FIGURE 3:**
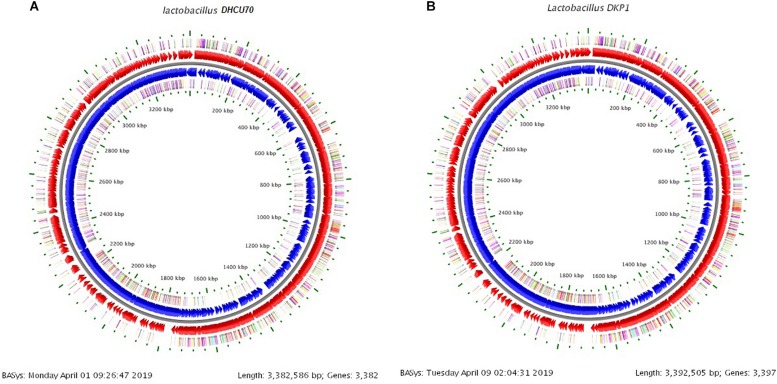
**(A)** Circular genome map of *Lactobacillus plantarum* DHCU70; **(B)** Circular genome map of *Lactobacillus plantarum* DKP1.

We obtained a complete genome sequence of *Lactobacillus plantarum* strains DHCU70 and DKP1, which showed a single circular chromosome (Figures 3A,B). Both strains shared maximum sequence similarity with *L. plantarum* strain WCFS1 (Kleerebezem et al., 2003) having a genome size of 3.38 and 3.39 Mb, and G + C content of 44.3%, respectively (Table 1). A total of 3252 and 3277 protein-coding sequences (CDSs) were identified in *L. plantarum* strains DHCU70 and DKP1, respectively, The 3191 and 3215 number of proteins present in strains DHCU70 and DKP1, respectively, were found to be functionally categorized among the predicted coding sequence for protein. Kleerebezem et al. (2003) have noted 3052 protein encoding sequences in *L. plantarum* strain Wcfs1. Both the strains of *L. plantarum* have no plasmid showing its significance in transformation and conjugation experiments in our study. Aukrust and Blom (1992) used *L. plantarum* and *L. sake* strains isolated from meat and vegetable for transformation. Similarly, Shrago et al. (1986) transferred erythromycin resistant streptococcal plasmid pAM β1 to *L. plantarum* via conjugation. The chromosome of *L. plantarum* strains DHCU70 and DKP1 contained 89 RNA genes including 69 tRNA, 16 rRNA and 4 ncRNA. Many strains of *L. plantarum* isolated from fermented vegetables and milk products have already been sequenced which include *L. plantarum* K25 from Tibetan Kefir (Jiang et al., 2018), *L. plantarum* SK151 from *kimchi* (Amoranto et al., 2018), and *L. plantarum* LL441 from dairy cheese (Flórez and Mayo, 2018) having genome size of 3.1 Mb (GC content of 44.6%), 3.2 Mb (GC content of 44.6%), 3.1 Mb (GC content of 44.5%), respectively. Zhang et al. (2009) reported 62 tRNA and 16 rRNA encoding genes in *L. plantarum* JDM1. However in our study we characterized bacteriocin encoding genes in *L. plantarum* strains DHCU70 and DKP1 through whole genome sequencing.

**TABLE 1:** Comparison of the features of *Lactobacillus plantarum* genome with reference genome.

Strain	*L. plantarum* DHCU70	*L. plantarum* DKP1	Reference strain (*L. plantarum* WCFS1)
Source	*Dahi*	*Kinema*	Human saliva
Genome Size(bp)	3383299	3393069	3308274 (Chromosomal DNA)
G + C Content (%)	44.3%	44.3%	45.6%
Total number of genes	3252	3277	3174
Coding genes	3191	3215	3063
Pseudogenes	61	62	23
Total no of RNA	89	89	88
No. of tRNA	69	69	70
No. of rRNA	16	16	15
No. of nc RNA	4	4	3
No. of repeat regions (CRISPER Array)	1	2	
No. of prophage region	6	7	4

Based on the phylogenetic analysis of 16S rRNA gene and *rec*A gene (Devi et al., 2016) through BLASTn, strains DHCU70 and DKP1 were found to display more than 99% similarity with *L. plantarum* group strains (Figure 1B). However, genomes of different *L. plantarum* group strains were difficult to distinguish by 16S rRNA gene sequence similarity since current taxonomy of *L. plantarum* group has closely related with species of *L. paraplantarum, L. pentosus, L. arixonensis, L. plantarum* subsp. *plantarum, L. plantarum* subsp. *argentoratensis, L. xiangfangensis*, and *L. fabifermentans* (Torriani et al., 2001; Kostinek et al., 2005; Devi et al., 2016). Phylogenetic tree was constructed based on the whole genome sequence to understand the phylogenetic relationship among *L. plantarum* strains which showed a close relationship with *L. plantarum* subsp. *plantarum* strain WCFS1 and strain MF1298 (Figure 1C). This observation suggests that the strains DHCU70 and DKP1 belonged to *L. plantarum* subsp. *plantarum*.

### Characterization of Bacteriocin Locus

**FIGURE 4:**
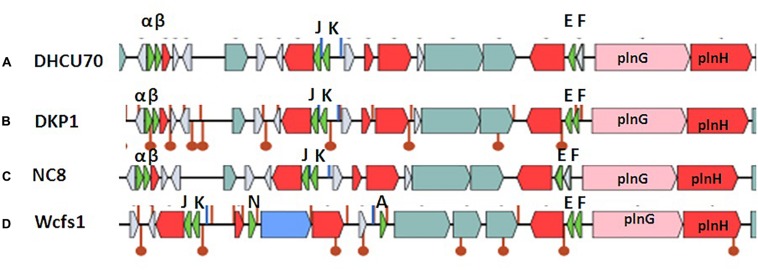
Genetic organization of *pln* locus of **(A)**: *Lactobacillus plantarum* DHCU70; **(B)** *L. plantarum* DKP1; **(C)**: *Lactobacillus plantarum* NC8; **(D)**: *Lactobacillus plantarum* WCFS1; Indicators: α – NC8α, β– NC8β, J – *pln* J, K- *pln*K, E-*pln*E, F-*pln*F, Red color indicated immunity protein.

Screening of the entire genome of *L. plantarum* strains DHCU70 and DKP1 revealed that bacteriocin encoding locus (*pln* locus) was located in a 20.5 kb long region consisting of 23 genes (*pln*) organized in operon-like structure (Figure 4). Strains DHCU70 and DKP1 have genes encoding two peptides plantaricin *pln*JK (classIIb), *pln*EF (classI) and inducible classII plantaricin NC8βα. The presence of two *pln* loci encoding plantaricins from two different classes contributes to a broad inhibitory spectrum of *L. plantarum* (Tai et al., 2015). Precursor peptides of NC8α and NC8β were made up of 47 and 55 amino acids, respectively, comprising leader sequences of the double-glycine type at N-terminal (Maldonado et al., 2003). However, the mature alpha and beta peptides contained 29 and 34 amino acids, respectively. Both the strains have a regulatory operon which included the inducing peptide encoding genes *pln*c8IF and histidine protein kinase *pln*c8K, as reported earlier in *L. plantarum* NC8 (Maldonado et al., 2003). The presence of plantaricin secretary genes *pln*G and *pln*H has also been confirmed in *L. plantarum* strains DHCU70 and DKP1 (Table 2), which are involved in the ABC transport system (Rizzello et al., 2014). The *pln* locus of the strains appeared to form several operons. Their production is regulated by secreted peptide pheromone, a membrane-located sensor and transcription regulators (Diep et al., 2009). Comparative analysis of *pln* locus with other known *pln* locus showed that both *L. plantarum* strains DHCU70 and DKP1 matched with class IIb two peptide antibiotics with *L. plantarum* NC8 as the closest neighbor (Axelsson et al., 2012).

**TABLE 2:** Comparison of *pln* genes of *Lactobacillus plantarum* strains DHCU70 and DKP1 with reference strains (NC8 and WCFS1).

*pln* genes	Function	*Lactobacillus plantarum* strains			
		DHCU70	DKP1	NC8	WCFS1
*pln*A	Induction pheromome	–	–	–	+
*pln*B	Histidine protein kinase	–	–	–	+
*pln*C	Response regulator	–	–	–	+
*pln*D	Response regulator	+	+	+ (99.6%)	+
*pln*EF	Prebacteriocin with GC leader	+	+	+(100%)	+
*pln*G	ABC Transporter	+	+	+ (99.7%)	+
*pln*H	Accessory protein	+	+	+(98%)	+
*pln*JK	Prebacteriocin with GC leader	+	+	+(100%)	+
*pln*L	Immunity protein	+	+	+ (100%)	+
*pln*MN	Prebacteriocin with GC leader	–	–	–	+
*pln*NC8α	Prebacteriocin with GC leader	+	–	+(100%)	–
*pln*NC8β	Prebacteriocin with GC leader	+	+	+(100%)	–
*pln*NC8IF	Induction pheromome	+	+	+(100%)	–
*pln*NC8HK	Histidine protein kinase	+	+	+(100%)	–

*Lactobacillus plantarum* strains were reported to have diversity in *pln* locus. *Lb plantarum* strain C11 is found to be organized in five operons-regulatory operon *pln*ABCD, *pln*EFI, *pln*JKLR, *pln*MNOP, *pln*GHSTUVWYXY (Daeschel et al., 1990). Whereas, organization of *pln* operon in *L. plantarum* strain NC8 was found to be similar to that of C11 with different regulatory operons consisting of *pln*NC8IF, *pln*NC8HK and *pln*D (Maldonado et al., 2004). *L. plantarum* type strains J23 and J51 also shows common feature of both strains C11 and NC8 with new ORFs (Rojo-Bezares et al., 2008; Navarro et al., 2008).

### Probiotic Characters

We analyzed various probiotic genes responsible for stress resistance, active removal of stressors, bile salt hydrolase activity, adhesion ability and immunomodulatory activity to find out the probiotic potential of bacteriocin producing *L. plantarum* strains DHCU70 and DKP1 (Table 3). Both *L. plantarum* strains DHCU70 and DKP1 were found to have *dlt*A&D and *gad*B genes, through whole genome sequencing analysis (Figure 3), which are responsible for acid tolerance, *bsh* gene for bile tolerance, *clp*L gene for acid and bile tolerance and *dlt*B gene for anti-inflammatory potential (Yunes et al., 2016; Bustos et al., 2018). We have also identified the genes Mucin22 and *fbp* responsible for adhesion ability to the intestinal epithelial layer (Turpin et al., 2012), probably to exclude the pathogenic species (Garcia-Gonzalez et al., 2018). Probiotics attributes shown by *L. plantarum* strains DHCU70 and DKP1 may be responsible for the potential capability to survive *in vitro* environmental stresses and *in vivo* human GIT conditions (Varankovich et al., 2015). *L. plantarum* strains isolated from different plant or animal (milk)-origins have antioxidant activity, probiotic effect, and protein fortification (Behera et al., 2018). Probiotic *Lactobacillus* shows probiotic factors such as stress response and adherence ability (Lebeer et al., 2008), and also to adaptation factors which include microbe-microbe interaction, epithelial barrier protection and immune modulatory effects (Llewellyn and Foey, 2017; Song et al., 2018).

**TABLE 3:** Probiotic related genes present in *Lactobacillus plantarum* strains DHCU70 and DKP1.

Gene	Putative function	Response	*Lactobacillus plantarum* strains	
			DHCU70	DKP1
**Stress resistance genes**				
*dlt*D (*L. rhamnosus*)	d- anylation of LTA	Acid and defensin Resistance	+	+
*dlt*A (*L. reuteri*)	d- anylation of LTA	Acid and defensin Resistance	+	+
**DNA and protein protection and repair**				
*dps*(*L. reuteri*)	DNA protection during starvation	DNA protection during starvation	+	+
*clp*L(*L. reuteri*)	*clp*ATPase (chaperon)	Acid and bile tolerance	+	+
*clp*C (*L. plantarum*)		Persistence capacity *in vivo*	+	+
*msr*B (*L. reuteri*)	Methionine sulfoxide reductase	Persistence capacity *in vivo*	+	+
*lux*S (*L. reuteri*)	Activated methyl cycle	Persistence capacity *in vivo*	+	+
**Active removal of stressors**				
*gad*B (*L. acidophilus*)	GABA transporter	Acid tolerance	+	+
*bsh* (*L. plantarum*)	Bile salt hydrolase	Bile salt resistance	+	+
**Anti-pathogenic effect**				
*lux*S (*L. reuteri*)	Production of AI-2. AI-3	Autoinduction ability	+	+
**Immunomodulation**				
*dlt*B (*L. plantarum*)	d- anylation of LTA	Anti-inflammatory potential *in vitro* in PBMCs and *in vivo* in a murine model of colitis or in a rat model for visceral pain perception	+	+
*dlt*D (*L. reuteri*)	d- anylation of LTA	Resistance to human β-defensin-2	+	+
**Adhesion ability**				
Mucin22	Mucin binding	Adhesion ability	+	+
*fbp*	Fibronectin binding	Adhesion ability	+	+

**FIGURE 5:**
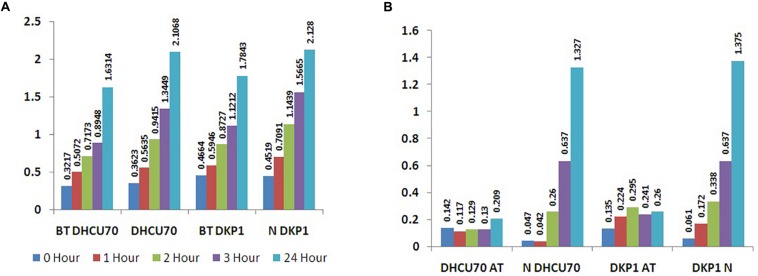
**(A)** Bile tolerance of *L. plantarum* strains DHCU70 and DKP1; **(B)** Acid tolerance of *L. plantarum* strains DHCU70 and DKP1.

*Lactobacillus plantarum* strains DHCU70 and DKP1 were subjected to acid tolerance at bile tolerance and pH3. Strains DHCU70 and DKP1 were found to have high tolerance rate to bile at 3 h (Figure 5A and Table 4) and pH 3 (Figure 5B and Table 4). Strain DHCU70 also showed growth in the given conditions to tolerate the stress till 24 h. The pH of human stomach varies from 1.5 (before food) to 5.0 (after food) due to the secretion of gastric juices (Cotter and Hill, 2003). Small intestine also receives various ranges of bile from liver (Staley et al., 2017). So, it is important for a probiotic bacterium to tolerate such conditions in order to survive in human GIT tract. Our findings showed that both the cultures are able to withstand the acidic and bile conditions of human GIT tract.

**TABLE 4:** Bile and acid tolerance of *L. plantarum* isolates DHCU70 and DKP1.

Treatments and strains	Hour (x 10^7^ cfu/ml)				
	0	1	2	3	24
**Bile tolerance:**					
DHCU70	1.6	1.8	2.1	2.2 (100%)^a^	2.7
DKP1	1.9	2.0	2.2	2.3 (100%)^a^	2.5
**Acid tolerance:**					
DHCU70	2.8	1.2	1.9	2.0 (71%)^a^	2.9
DKP1	2.7	1.5	1.8	1.9 (65%)^a^	1.9

*^*a*^Percentage (%) survival.*

*Lactobacillus plantarum* strains DHCU70 and DKP1 were analyzed for antioxidant activity through DPPH radical-scavenging assay and showed 77.57 and 68% antioxidant activity (Supplementary Table S2a), respectively. Many *Lactobacillus* strains isolated from fermented foods were reported to have antioxidant activity ranging from 40 to 90% (Kuda et al., 2014). Such probiotic isolates with high antioxidant ability helps to reduce the oxidative stress (Li et al., 2012). This suggests that our *L. plantarum* cultures can be used as a suitable probiotic candidate in food industry. Bacterial adhesion was evaluated using hydrocarbons like xylene and *n*-hexadecane. Auto-aggregation ability of strain DHCU70 was 72.84% whereas for strain DKP1 was 52.91% (Supplementary Table S2b). The percentage hydrophobicity for *L. plantarum* strains DHCU70 and DKP1 was 55.57% and 5.40% for xylene, respectively, and 42.9 and 40.88% for hexadecane, respectively (Supplementary Table S2c). *L. plantarum* shows a defense mechanism against the pathogen due to its hydrophobic and auto aggregative ability (Honey Chandran and Keerthi, 2018).

## Conclusion

*Lactobacillus plantarum* strains DHCU70 and DKP1 isolated from fermented foods *dahi* and *kinema*, respectively, found to have inducing peptides, immunity peptide and ABC transporter proteins. Originally analyzed *L. plantarum* strain NC8 having NC8-type bacteriocin was isolated from grass silage. However *L. plantarum* DHCU70 having the same NC8 type of bacteriocin has been isolated from dairy origin, *dahi* (fermented milk product) which indicates that *L. plantarum* DHCU70 of dairy origin may have better adaptability to the GIT conditions. Moreover, the mode of action studies showed that both the strains of *L. plantarum* DHCU70 and DKP1 have novel mode of actions that may help in resolving the problem of antibiotic resistance. Along with antibacterial properties, strains *L. plantarum* DHCU70 and DKP1 are found to have probiotic genes which may help them to survive *in vitro* environmental stresses and *in vivo* human GIT conditions, indicating as potential probiotic candidates.

## Data Availability Statement

Genome sequence of the *L. plantarum* strains DHCU70 and DKP1 was submitted to NCBI under accession numbers RZJQ00000000 and SDIE00000000, respectively.

## Author Contributions

AG, PH, and JT formulated and designed the study. AG and PH performed, analyzed, and interpreted the experiments along with the preparation of the manuscript draft. JT and PH have finalized the manuscript for publication.

## Conflict of Interest

The authors declare that the research was conducted in the absence of any commercial or financial relationships that could be construed as a potential conflict of interest.

## Footnotes

1 www.fda.gov

2 www.efsa.europa.eu

## Supplementary Material

FIGURE S1 Antimicrobial activity under various environmental conditions. **(a)** AMA at pH 1, **(b)** AMA at pH 9, **(c)** AMA at 121°C, and **(d)** AMA of PK treated CFS.

TABLE S1 AMA under various environmental conditions.

TABLE S2 **(a)** Antioxidant activity (DPPH Assay), **(b)** Autoaggregation, and **(c)** Hydrophobicity.
