# Supplementary figures and images for: Genome Analysis of Lactobacillus plantarum Isolated From Some Indian Fermented Foods for Bacteriocin Production and Probiotic Marker Genes
Source: Front Microbiol. 2020 Jan 29;11:40. doi: 10.3389/fmicb.2020.00040 (PMC7000354; doi:10.3389/fmicb.2020.00040)

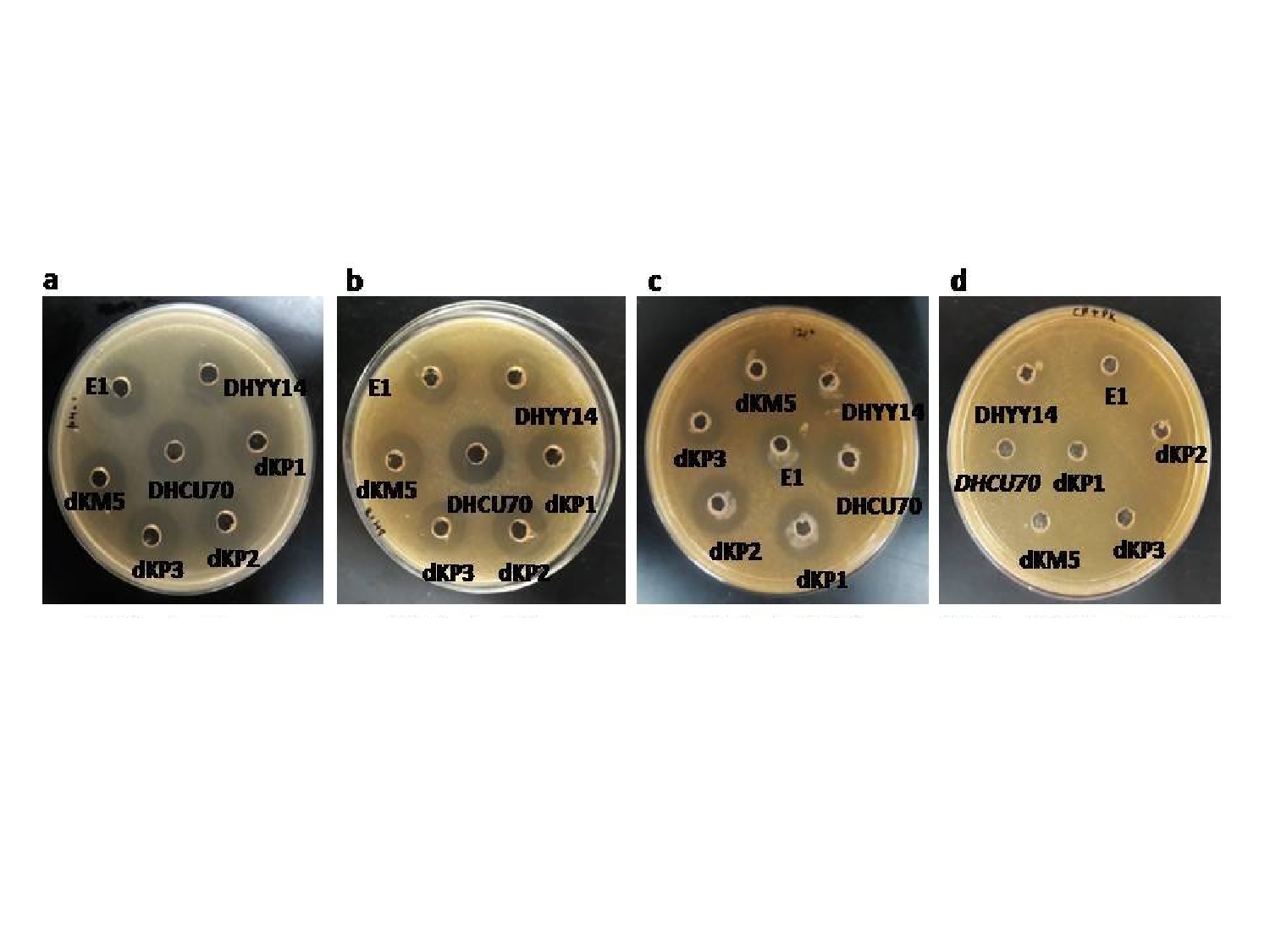

Supplement: FIGURE S1 — Antimicrobial activity under various environmental conditions. (a) AMA at pH 1, (b) AMA at pH 9, (c) AMA at 121°C, and (d) AMA of PK treated CFS. [file Image_1.jpg]
