# Supplementary material for: Genome Analysis of Lactobacillus plantarum Isolated From Some Indian Fermented Foods for Bacteriocin Production and Probiotic Marker Genes
Source: Front Microbiol. 2020 Jan 29;11:40. doi: 10.3389/fmicb.2020.00040 (PMC7000354; doi:10.3389/fmicb.2020.00040)
Supplement: TABLE S1 — AMA under various environmental conditions. [file Table_1.pdf]

Table 1: AMA under various environmental conditions

	CFS	CFS+PK	Temperature				pH		
			60°	80°	100°	121°	pH9	pH3	pH7
**dKP1**	20	NZ	19	19	19	20	19	20	15
**dKP2**	18	NZ	18	18	18	19	18	19	18
**dKP3**	18	NZ	18	18	16	16	18	19	18
**dKM5**	18	NZ	18	18	16	16	18	19	19
**DHCU70**	20	NZ	19	19	19	20	19	21	18
**DHYY14**	18	NZ	17	17	16	16	18	19	17
**E1**	17	NZ	17	17	15	15	18	19	17
