# Supplementary material for: Genome Analysis of Lactobacillus plantarum Isolated From Some Indian Fermented Foods for Bacteriocin Production and Probiotic Marker Genes
Source: Front Microbiol. 2020 Jan 29;11:40. doi: 10.3389/fmicb.2020.00040 (PMC7000354; doi:10.3389/fmicb.2020.00040)
Supplement: TABLE S2 — (a) Antioxidant activity (DPPH Assay), (b) Autoaggregation, and (c) Hydrophobicity. [file Table_2.pdf]

Table2a: Antioxidant activity(DPPH Assay)			
	OD(Control)	OD TEST	Result
DHCU70	2.3413	0.5158	77.96%
DKP1	2.3413	.736	68%

DPPH radical scavenging activity % = [(Acontrol–Atest) /Acontrol] x 100

Table2b: Autoaggregation			
	OD (Total)	OD (Upper suspension	Result
DHCU70	.7401	.2011	72.84%
DKP1	.2576	.1213	52.9%

Percentage aggregation = 1–(OD of upper suspension/OD of total culture)×100

Table2c: Hydrophobicity						
	Xylene			N- hexadecane		
	OD(Initial)	OD Final	Result	OD(Initial)	OD Final	Result
DHCU70	.7001	.3111	55.57%	.6481	.37	42.9%
DKP1	.7400	.3670	50.40%	.7777	.46	40.88%

Surface Hydrophobicity = 100 × (AbI-AbF)/ AbsInitial
